# Immune Thrombocytopenic Purpura following Administration of mRNA-Based SARS-CoV-2 and MMR Vaccinations: A Cautionary Tale

**DOI:** 10.1155/2021/2704249

**Published:** 2021-10-09

**Authors:** Whitney Thomas, Adam Albano, Dean Kirkel, Nason Rouhizad, Folasade Arinze

**Affiliations:** ^1^Department of Internal Medicine, Wellstar Health System, Marietta, GA, USA; ^2^Northwest Georgia Oncology Centers, P.C Wellstar Health System, Marietta, GA, USA

## Abstract

We report a case of immune thrombocytopenic purpura (ITP) in an otherwise healthy 31-year-old man following coadministration of the live measles, mumps, and rubella (MMR) vaccine with the Pfizer-BioNTech mRNA SARS-CoV-2 vaccine. The patient was hospitalized briefly and treated for ITP with glucocorticoids, IVIG, and platelet transfusion. Although our patient's clinical presentation and subsequent course are similar to those of other cases of ITP in association with SARS-CoV-2 vaccination, to our knowledge, this is the first reported case of ITP following MMR and mRNA SARS-CoV-2 vaccine coadministration. It would be impossible to conclusively prove that the patient's thrombocytopenia was secondary to the SARS-CoV-2 vaccine alone, the MMR vaccine, or an additive effect of both vaccines. However, with the CDC guidelines recommending the coadministration of the mRNA SARS-CoV-2 vaccine without regards to timing with other vaccines, we urge further caution as there is limited evidence to inform practice. This case highlights the need for further safety data regarding the coadministration and timing of the mRNA SARS-CoV-2 vaccine with other vaccines.

## 1. Introduction 

The Centers for Disease Control guidelines recommend the coadministration of COVID-19 vaccines and other vaccines without regard to timing [1]. Recent reports of patients developing ITP following SARS-CoV-2 mRNA and adenovirus vaccinations have raised concerns of a possible relationship between vaccine administration and the development of ITP, although a causal role has not yet been established with certainty [2]. This association is of particular importance with the coadministration of COVID-19 and the MMR vaccines because MMR vaccine-induced ITP has been well established. There are currently no data about the incidence of ITP following coadministration of MMR and SARS-CoV-2 mRNA vaccines [3].

We describe a case of ITP developing shortly after receiving the Pfizer-BioNTech vaccine series in a patient who had also received a first dose of the MMR vaccine series 6 weeks prior to the second mRNA SARS-CoV-2 vaccine.

## 2. Case Presentation

A 31-year-old man with no significant past medical history presented to an urgent care center with complaints of fatigue, rash, and epistaxis. Two and a half weeks prior, he received his second scheduled dose of the Pfizer-BioNTech mRNA SARS-CoV-2 vaccine. Within twenty-four hours of vaccine administration, he developed fatigue and a petechial rash across his waistband. Symptoms worsened over the next several weeks, and he noted easy bruising as well as additional petechial lesions. Of note, the patient had received the first scheduled dose of the two-dose live measles, mumps, and rubella (MMR) vaccine series exactly three and six weeks prior to receiving the first and second doses of the Pfizer-BioNTech mRNA SARS-CoV-2 vaccinations, respectively. Complete blood count showed a white cell count of 5.1 × 10^9^/L, hemoglobin 14.8 g/dL, and platelet count of 1,000/*µ*L, and he was subsequently admitted to hospital for additional evaluation and management.

**Figure 1:**
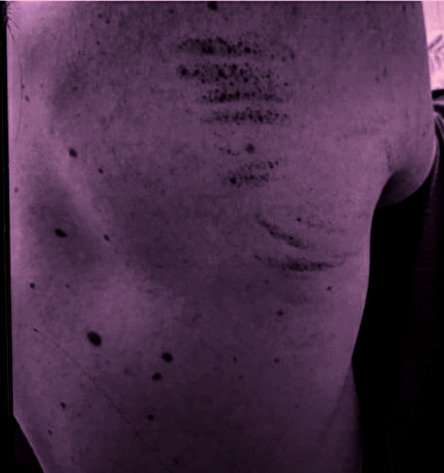
Petechial rash along the right scapula.

**Figure 2:**
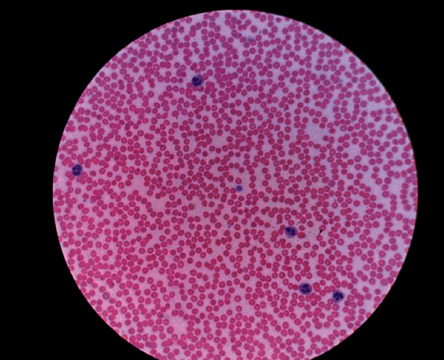
Peripheral smear with rare, large platelets.

Physical examination was notable for a well-appearing young male with normal vital signs. Skin examination revealed a scattered petechial rash along the right scapular area of the patient's back and across the patient's waistline (Figure 1). The remainder of his exam was unremarkable. Additional laboratory testing showed a normal lactate dehydrogenase of 173 IU/mL, haptoglobin 22 mg/dL, and fibrinogen 233 mg/dL. Testing for HIV and hepatitis C was negative. Vitamin B12 and folate were within the normal range. Review of the peripheral blood smear was notable for markedly reduced platelet count with rare, large circulating platelets. No schistocytes or other distinctive erythrocyte abnormalities were identified (Figure 2). A diagnosis of immune thrombocytopenia purpura (ITP) was made, and the patient was treated with intravenous immune globulin 1 g/kg and glucocorticoids. He was also transfused 2 units of platelets. Platelet count improved significantly to 95,000/*µ*L by hospital day 2, and he was subsequently discharged home. Repeat measurement of his platelet count 3 days after discharge was 472,000/*µ*L. Glucocorticoids were discontinued, and he remained with normal hematologic parameters at 5 weeks after diagnosis.

## 3. Discussion

### 3.1. The Causative Role of the MMR Vaccine in the Development of ITP

The underlying mechanism of SARS-CoV-2 vaccine-associated ITP remains unknown, but several theories rest upon knowledge from MMR vaccine-induced ITP. The development of ITP after MMR administration was first described in 1966, and additional cases have been documented since then [4]. The causal relation of the MMR vaccine with ITP involves a mechanism that includes the development of autoantibodies cross reacting with antigenic targets on platelets. In a study of ITP developing after MMR vaccination, 79% of patients had antibody-coated platelets targeted at GP Ib/IX, GP Ia/IIa, and GP VI.10 antigens [4].

ITP following MMR vaccination occurs more frequently in children because their developing idiotypic immune network has an increased likelihood of postvaccination cross reactivity [4]. According to the CDC, the risk of ITP is the highest in the six weeks following vaccine administration and occurs at a rate of approximately 1 : 40,000 vaccinations [1]. The disease is usually self-limited with mild to moderate thrombocytopenia [5].

Our patient received the first dose of the MMR vaccine series exactly six weeks prior to his second dose of the Pfizer mRNA SARS-CoV-2 vaccine. The onset of his petechial rash occurred within the six-week window of elevated risk that has been classically described with MMR vaccine-induced ITP. Nevertheless, whether the patient's ITP developed as a consequence of the MMR vaccine, the mRNA SARS-CoV-2 vaccine, or both cannot be proven. The patient did not receive lab work in the interim period between his MMR vaccination and his two subsequent Pfizer vaccinations, and it is unclear when his platelet levels initially began to fall.

### 3.2. The Association of SARS-CoV-2 Vaccination with Immune Thrombocytopenia

With the historically rapid development of the SARS-CoV-2 vaccines in response to the global pandemic, there has been increased vigilance around vaccine administration and its untoward effects. Concerns over a possible association with the development of ITP and mRNA SARS-CoV-2 vaccine were already emerging before the highly publicized account of ITP, brain hemorrhage, and subsequent death of a previously healthy 56-year-old man sixteen days following Pfizer vaccination [6]. Other incident cases of ITP after the administration of the mRNA SARS-CoV-2 vaccines continue to be reported in the Vaccine Adverse Event Reporting System (VAERS). A review of pharmacovigilance databases at VAERS (last accessed 7/25/21) has shown at least 744 cases of thrombocytopenia or immune thrombocytopenia following mRNA SARS-CoV-2 vaccine administration in the United States [7]. The one confirmed death is currently still under investigation [6].

Despite the number of reported cases of SARS-CoV-2 vaccine-induced ITP and ongoing investigations of this association, a causal role is yet to be identified. Most recently, the AstraZeneca adenovirus vaccine was shown to have an association with the development of both thrombotic and hemorrhagic platelet dysfunction, including vaccine-induced thrombotic thrombocytopenia (VITT) as well as ITP [8]. Simpson et al. implicated the AstraZeneca adenovirus vaccine in the development of ITP in a Scottish prospective case control series, and an additional 1.13 cases of ITP were estimated to occur per 100,000 doses of the adenovirus vaccine [9]. Of note, however, the authors did not find an association between ITP and the first dose of the Pfizer mRNA SARS-CoV-2 vaccine [9].

Mechanistically, the fact that the novel vaccines induce protein synthesis is important. As far back as the 1960s, investigations have shown that platelets are capable of synthesizing proteins [10]. This ability of platelets to synthesize proteins not only alters their phenotype and function, but also theoretically allows platelets to synthesize the immunogenic spike protein from the mRNA SARS-CoV-2 vaccines [2, 10]. The COVID-19 vaccines, therefore, could trigger a platelet-directed immune response through mRNA translation and resultant spike protein synthesis. The subsequent immune response to the spike proteins of the platelets could then generate immune thrombocytopenia. Other possible mechanisms were summarized by Lee et al. (1) an RNA-generated immune response from dendritic cells, (2) preformed antibodies to vaccine components, (3) preexisting ITP or thrombocytopenia in the individual cases, and (4) postvaccination ITP [2].

Establishing a causal role between the administration of SARS-CoV-2 vaccines and the development of ITP would be challenging due to the lack of prevaccination platelet counts and the variable time after vaccination to the appearance of thrombocytopenia. Additionally, ITP is a diagnosis of exclusion; there is no specific laboratory test to confirm the diagnosis. It remains to be seen if the incidence of immune-related thrombocytopenia following SARS-CoV-2 vaccination is comparable to background risk.

While proving an association between ITP and the COVID-19 vaccines is still under investigation, coadministration of mRNA SARS-CoV-2 vaccines with other vaccines and the development of ITP remains completely unexplored. This is the first reported case of ITP following MMR and mRNA SARS-CoV-2 vaccine coadministration. It is unknown if there is an elevated risk of developing ITP following the coadministration of the two vaccines, and it would be prudent to obtain further data to ensure that no relative contraindications exist.

## 4. Conclusions

Our case serves as an example of an emerging association with immune thrombocytopenia, and the need for continued vigilance as SARS-CoV-2 vaccination becomes more widespread. Further analysis is warranted, and review of the VAERS should be ongoing. As the FDA expands the use of Pfizer mRNA SARS-CoV-2 vaccination to children, who are historically more likely than adults to develop ITP from the MMR vaccine [11, 12], we emphasize the need for additional vigilance with respect to ITP which will provide data for formal recommendations regarding the appropriate time interval between mRNA SARS-CoV-2 and the MMR vaccines in both children and adults. This information will be useful for governmental organizations such as the Centers for Diseases and Control as they offer guidance regarding the timing of administration of SARS-CoV-2 vaccines and other immunizations.

## Data Availability

The incidence of vaccine-related thrombocytopenia and immune thrombocytopenia purpura that is cited in this case report is available in/from the Vaccine Adverse Event Reporting System (VAERS), https://vaers.hhs.gov.
